# Systems Pharmacology for Investigation of the Mechanisms of Action of Traditional Chinese Medicine in Drug Discovery

**DOI:** 10.3389/fphar.2019.00743

**Published:** 2019-07-11

**Authors:** Wenjuan Zhang, Ying Huai, Zhiping Miao, Airong Qian, Yonghua Wang

**Affiliations:** ^1^Lab for Bone Metabolism, Key Lab for Space Biosciences and Biotechnology, School of Life Sciences, Northwestern Polytechnical University, Xi’an, China; ^2^Research Center for Special Medicine and Health Systems Engineering, School of Life Sciences, Northwestern Polytechnical University, Xi’an, China; ^3^NPU-UAB Joint Laboratory for Bone Metabolism, School of Life Sciences, Northwestern Polytechnical University, Xi’an, China; ^4^Lab of Systems Pharmacology, College of Life Sciences, Northwest University, Xi’an, China

**Keywords:** bioactive compounds, target identification, systems pharmacology, synergistic effect, drug discovery

## Abstract

As a traditional medical intervention in Asia and a complementary and alternative medicine in western countries, traditional Chinese medicine (TCM) has attracted global attention in the life science field. TCM provides extensive natural resources for medicinal compounds, and these resources are generally regarded as effective and safe for use in drug discovery. However, owing to the complexity of compounds and their related multiple targets of TCM, it remains difficult to dissect the mechanisms of action of herbal medicines at a holistic level. To solve the issue, in the review, we proposed a novel approach of systems pharmacology to identify the bioactive compounds, predict their related targets, and illustrate the molecular mechanisms of action of TCM. With a predominant focus on the mechanisms of actions of TCM, we also highlighted the application of the systems pharmacology approach for the prediction of drug combination and dynamic analysis, the synergistic effects of TCMs, formula dissection, and theory analysis. In summary, the systems pharmacology method contributes to understand the complex interactions among biological systems, drugs, and complex diseases from a network perspective. Consequently, systems pharmacology provides a novel approach to promote drug discovery in a precise manner and a systems level, thus facilitating the modernization of TCM.

## Introduction

Traditional Chinese medicine (TCM) plays important roles in the prevention and treatment of complex diseases, which has been developed in China for thousands of years (Tang et al., 2009). In recent decades, TCM has been widely used as the complementary and alternative medicine in Western countries. Generally, Chinese herbal prescriptions or formulae (also called “Fangji”) are used in clinical practice, and they can exhibit coordinating roles through the rational combination of multiple herbs to achieve good efficacy and few side effects for various diseases’ prevention and treatment (Li et al., 2009). Despite the widespread use of TCM in clinical practice, proving its effectiveness *via* scientific trials and dissecting the molecular mechanisms are still big challenges.

Indeed, TCM and Chinese medicine formulae are designed under the principle of “syndrome differentiation” according to the combination rule of medicinal properties in TCM with obvious multiple-compound characteristics (Li, 2009). In ancient times, ancestors usually tested poison to identify effective herbs; for example, Li Shizhen was a famous physician and pharmacologist in the Ming Dynasty, who tested drugs and tried poison in the spirit of dedication to science. There is no doubt that viewing humans as the testers would be risky, but knowledge on common herbs is the achievement of an Ancient Chinese medical scientist who tried poison. Nowadays, great efforts have been made to extract and isolate compounds in herbs and prescriptions, resulting in the emergence of numerous newly identified ingredients (Zhang et al., 2018). In addition, the absorption, distribution, metabolism, and excretion (ADME) properties are defined as the dynamic changes in drugs within an animal or the human body, such as oral bioavailability (OB), drug-likeness, and half-life, which are critical in drug discovery and development (Su et al., 2007). It has been reported that nearly 95% of lead compounds fail in the drug development in clinical trials each year, and approximately 50% of these failures are due to poor ADME properties (Kassel, 2004). Therefore, the optimization of the ADME properties of lead compounds may be a critical factor that determines whether the drug can be successfully developed (MacCoss and Baillie, 2004). Many clinical studies including randomized controlled trials (RCTs) of the herbs have been conducted, some demonstrating hepatotoxicity and toxicity (Hu et al., 2017). However, because the extraction and isolation of compounds derived from herbs are costly and time-consuming, as well as only a few of them have satisfactory ADME properties and less side effects, there is an urgent need to develop a fast and effective novel strategy for identifying potential active compounds.

Furthermore, the identification of compounds derived from TCM is also an important process for drug development and an essential factor for the dissection of the holistic mechanisms of action of TCM (Cao et al., 2012). Currently, the ligand-based virtual screening (LBVS), structured-based virtual screening (SBVS), and the text mining-based approach are widely used to predict the target–ligand interactions (Ballesteros and Palczewski, 2001; Byvatov et al., 2003; Krejsa et al., 2003). In addition, several chemical genomics approaches, such as the ligand-based, target-based, or target–ligand methods, are more effective to predict the compound–protein interactions (Balakin et al., 2003; Frimurer et al., 2005; Nagamine and Sakakibara, 2007; Rognan, 2007; Xia et al., 2009; He et al., 2010; Yamanishi et al., 2011). For example, Frimurer et al. have established a target-based approach to divide the receptors and the known ligands into clusters and further to discover each cluster with shared ligands (Frimurer et al., 2005). However, the target–ligand approach integrates the ligand chemical space, target space, and the available known drug–target network information to construct a complex predictive model to predict ligands or targets. For example, the *in silico* models integrated the amino acid sequences, two-dimensional chemical structures, and mass spectrometry data, as well as the chemical functional groups and biological features, for predicting the drug–target interactions (Nagamine and Sakakibara, 2007). However, all these approaches only focused on limited receptor space with certain protein families or the limited chemical space of US Food and Drug Administration (FDA)-approved drugs, and maybe they are not suitable for the unknown compounds of TCM. Therefore, novel approaches to identify the drug targets of TCM are valuable for understanding the mechanisms of TCM.

More importantly, TCM views the human body as a complex dynamical system and focuses on the balance of the human body, both internally and with its external environment (Ma et al., 2016). Previously, the researchers could only focus on the human body’s reaction to herbal medicines, such as alleviating cough, reducing heat, and limiting bleeding. However, how these active molecules combine with each other to assemble as a whole to exert their therapeutic effects is still unclear, and it is of great significance to understand the molecular mechanisms of TCM. Therefore, efficient approaches to dissecting the mechanisms of drug combinations in TCM are of great significance to understand the underlying mechanisms of action of TCM.

Fortunately, the advent of systems pharmacology has provided the opportunity and methodologies for the development and modernization of TCM. In the recent year, systems pharmacology has been used to identify active natural products and investigate the mechanism of natural products (Li et al., 2015a, Li et al., 2012b; Zhang et al., 2016; Fang et al., 2017; Wang et al., 2017; Yang et al., 2017). Also, systems pharmacology provides new strategy for discovering novel drug combinations for the treatment of complex diseases. Integrated TCM for treatment of various diseases based on syndrome differentiations is one essential factor of the compatibility principles contributing to the drug efficacy (Zhang and Wang, 2015; Wang and Li, 2016; Zhu et al., 2018). However, in contrast to Western medicine, TCM is overly dependent on the experiences of patients and practitioners and lacks systematic research methods. Therefore, there are many issues that need to be resolved in the development of TCM, for example: 1) TCM focused on the overall efficacy and clinical safety, but there is a lack of precise analysis and monitoring, including few studies on the pharmacodynamic and toxicological mechanisms; 2) the quality of herbs is one of the most important factors for the modernization of TCM, which has a major effect on the efficacy of TCM, but the quality is difficult to control; 3) the synergistic, additive, or antagonistic effects of TCM depend on the different properties of absorption, distribution, metabolism, excretion, and the toxicity of the pharmacodynamic components, which remain unclear; 4) the active ingredients and the mechanisms of action of TCM are unclear, which restrict the acceptance and development of TCM and seriously hinder the modernization processes. Owing to its complex composition and multiple systems, it is difficulty to dissect the underlying mechanisms of TCM at the systems level. Furthermore, the methodology often leads to controversy. Therefore, there is an urgent need to develop a new systematic and holistic research method.

In this review, we first introduced the concept and principle of systems pharmacology, and then we reviewed the computational methods of systems pharmacology for bioactive compound screening, target fishing, drug combination, and network analysis. In addition, we detailed the applications of systems pharmacology, including the elucidation of the mechanisms of action of herbal formulae, the design of multi-target drugs or drug combinations, and the theoretical analysis of Chinese medicine to guide the development of herbal medicine.

## Concept and Principle of Systems Pharmacology

**Figure 1:**
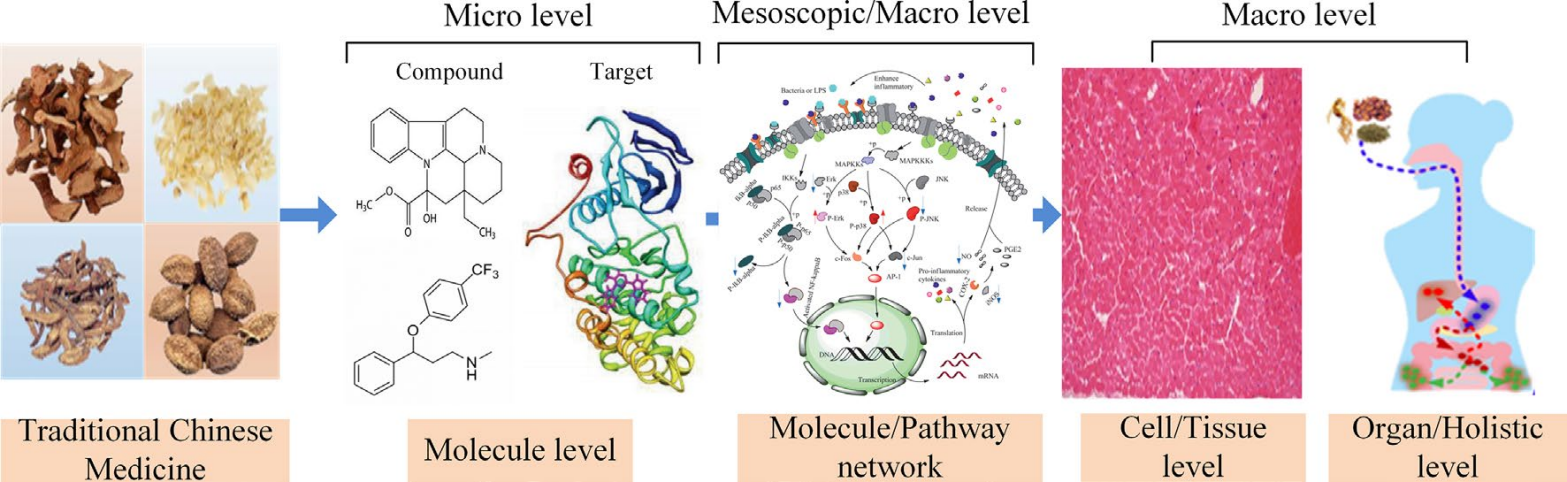
Multi-scale models and approaches of systems pharmacology.

The exploration of the mechanism of action of the multiple compounds within a TCM prescription is the inevitable requirement for the modernization of TCM. In addition, to uncover the mechanism of actions of TCM, the modern scientific and technological methods need to propose for the foundation to promote the global development of TCM. Owing to its complexity, the holistic concept, and syndrome differentiation of TCM theory, the dissection of mechanisms of action of TCM is difficult. Therefore, we proposed the systematic research approach of systems pharmacology, based on the dynamic interaction of TCM with the human body from different levels, such as cellular, molecular, tissue, organ, and holistic levels (Wang and Yang, 2013) (**Figure 1**).

Systems pharmacology is an emerging discipline that focuses on the interaction between drugs and the body and the rules and mechanisms of drugs at a systems level. More specifically, the interactions between drugs and the body are illustrated from the microscopic levels (molecular and biochemical network levels) to the macroscopic levels (tissue, organ, and holistic levels). Systems pharmacology aims to investigate the changes in the functions and reactions in the human body induced by drugs, thus providing new strategies and tools to achieve precise control of the complex biological networks inside cells, thus altering disease pathophysiology, improving drug efficacy, and reducing adverse reactions (Wang and Yang, 2013; Zhang and Wang, 2015). To enhance the systems pharmacology platform, theoretic calculations and experimental methods were integrated into the models for the discovery of bioactive molecules, the identification of new drug targets, the prediction of adverse drug reactions, the exploration of therapeutic mechanisms, and the elucidation of the rules of drug combination (Huang et al., 2013a). This platform allows the large-scale analysis of simulation methodology and optimization algorithms, which can be applied to determine the molecular mechanisms of TCM and to assist the development of novel drugs.

## Methodology of Systems Pharmacology in TCM

### ADME Screening Methods of Bioactive Ingredients in TCM

**Figure 2:**
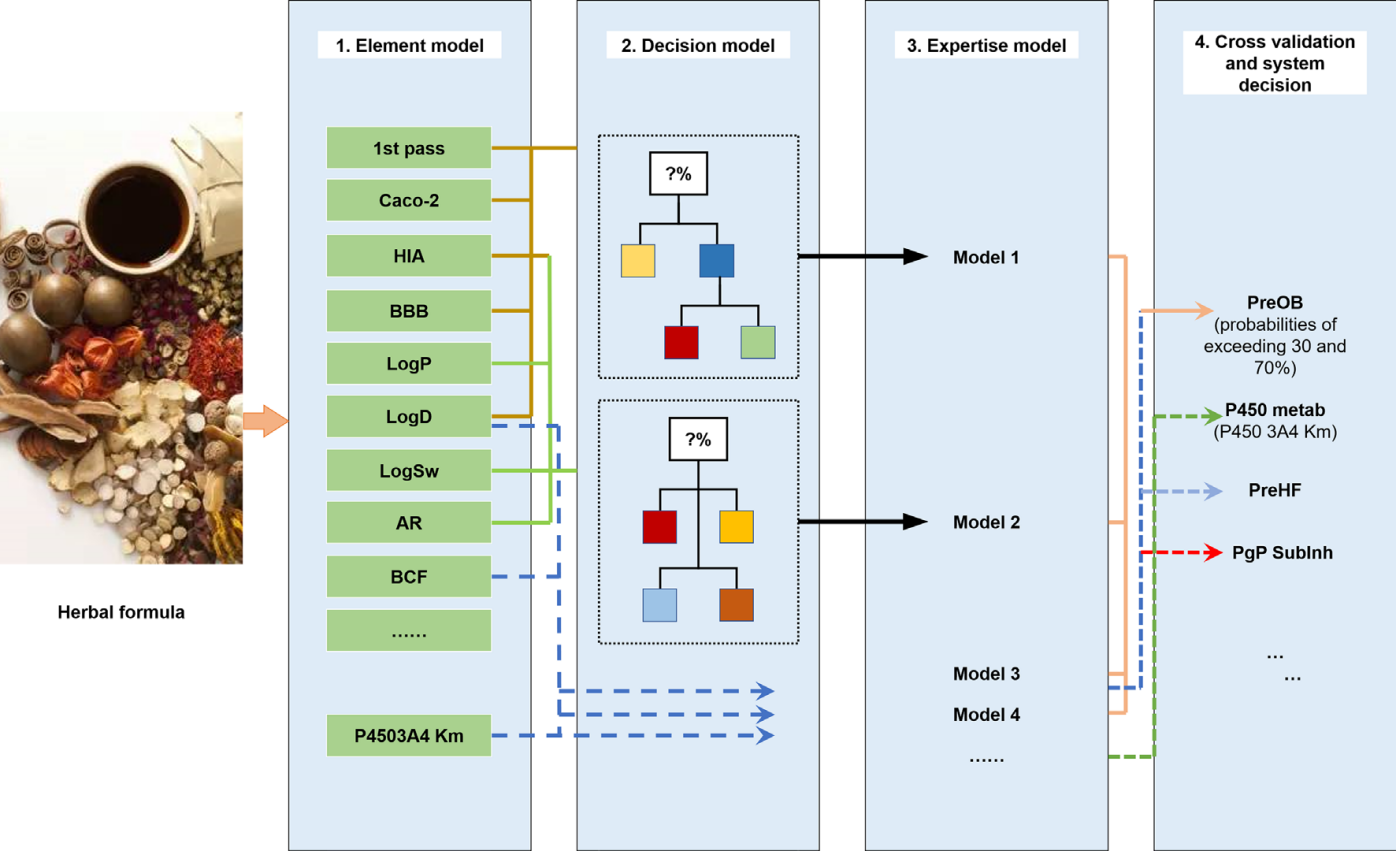
SysADME/t system for the screening of bioactive ingredients of traditional Chinese medicines (TCMs).

The ADME properties consist of drug solubility, permeability, protein binding ability, oral bioavailability, drug-likeness, blood–brain barrier (BBB) permeability, small intestine absorption, and half-life. TCM is a multifaceted system consisting of numerous compounds, of which only a few exhibit favorable ADME properties. Therefore, the screening and analysis of bioactive components in TCM are extremely challenging. To solve this problem, in the following section, we have focused on the introduction of an *in silico* ADME system (SysADME) (**Figure 2**), which is a rapid, efficient, and cost-effective strategy to explore the potential bioactive compounds of herbal medicines.

First, from the structure of the compounds and the help of system theory and artificial intelligence, the SysADME system integrates more than 20 models, including P-glycoprotein substrate inhibitor (Pgp) recognition, small intestine absorption, BBB permeability, and a mathematical forecast of plasma protein binding (Ai et al., 2009; Wang et al., 2009; Ai et al., 2010). In addition, we have built a series of predictive toxicity analysis (toxicology) models through the integration of modern statistics, chemical informatics, and other techniques (Hao et al., 2011; Xu et al., 2011a; Xu et al., 2011b). In the following part, we will review three representative models in details.

#### The Prediction of Human Oral Bioavailability (OB)

Because the predominant and most convenient way to deliver drugs of TCM is the oral route, the good OB of a new drug candidate is one of the essential pharmacokinetic parameters of ADME properties. Recently, multiple large-scale experiments have been conducted to evaluate the OB values of drugs, but they are labor-intensive and time-consuming. At first, Lipinski’s “rule of five” has been qualitatively used to predict the absorption and permeability of drugs to guide the prediction of OB (Lipinski et al., 2012). And then many *in silico* models have been established to predict OB of drug molecules in the early stages of drug discovery (Aller et al., 2009). Quantitative structure–property relationship (QSPR), rule of thumb (RoT), and physiologically based-pharmacokinetic (PBPK) approaches are promising alternatives to the OB prediction (Agoram et al., 2001; Cabrera-Pérez et al., 2018). Since 2000, numerous QSPR models have been developed to predict OB; for example, Andrews et al. constructed a regression model to predict OB based on a dataset of 591 molecules by applying 85 structural descriptors (Aller et al., 2009). Compared to Lipinski’s “rule of five,” the false-negative rate was reduced from 5% to 3%, and the false-positive rate decreased from 78% to 53%. In addition, Yoshida et al. used the multiple linear regression model for predicting OB with 15 structural descriptors (Yoshida and Topliss, 2000). However, the correct accuracy of this model can only achieve 60% for the test compounds. As for PBPK models, Yu and Amidon have established a compartmental model of absorption and transit (CAT) to predict the fraction of absorbed dose of different drugs (Yu, 1999). These integrated models were established based on seven transit compartments, which represent different anatomical regions of the small intestine. The limitation of the CAT model is that it ignored several properties that affect drug absorption, such as rate of dissolution, pH dependence on drug solubility, absorption in the stomach and/or colon, first-pass metabolism, and drug degradation in the intestine and liver, leading to the prediction of absorption with low solubility or permeability (Cabrera-Pérez et al., 2018). Up to now, there are no reliable and efficient models for prediction of OB based on simple descriptors.

In our previous work, given the multiple compounds, multiple targets, and synergetic effects of TCM, we have proposed a mathematical model called prediction of oral drug bioavailability (PreOB), which integrated the effects of Pgp efflux and P450 metabolism to ensure the accuracy of OB prediction of drugs (Xu et al., 2012). The PreOB was carried out by the following steps: first, 805 drug and drug-like molecules and their OB values were collected from the bioavailability database (Hou and Xu, 2002), and all the OB values were transformed into the common logarithm of log (oral bioavailability) (logB). Besides, a total of 1,536 dragon descriptors were calculated by Professional 5.4, 2006 (Talete, 2011). Then, all the 805 drugs were divided into several statistical subsets according to the geometry-based algorithm and iterative self-consistent approach (Jain, 2003). Next, by self-organizing map (SOM) (Vesanto, 2002), the compounds in each subset were split into training and independent validation sets based on their distribution in the chemical space. The two linear methods including multiple linear regression (MLR) and partial least squares regression (PLS), and the non-linear method support vector regression model (SVR) were available to perform prediction with five-fold cross-validation and independent external tests. The results showed that all the performance of SVR is slightly better than that of MLR and PLS, with its determination coefficient (*R* ^2^) of 0.80 and standard error of estimate (SEE) of 0.31 for test sets. The prediction abilities of the MLR and PLS are relatively weak, exhibiting 0.60 and 0.64 for the training set with SEE of 0.40 and 0.31, respectively. Our results showed that MLR-, PLS-, and SVR-based *in silico* models have good potential in the prediction of OB and may facilitate the drug design. Generally, the compounds meeting the criteria of OB ≥ 30% are considered as potential active compounds with satisfactory pharmacological properties. The comparisons between the tools of the prediction of OB developed by the other groups and PreOB model are summarized in **Table 1**. More importantly, the PreOB model has been successfully applied for material-based analysis of many Chinese medicines (Li et al., 2012b; Liu et al., 2013a).

**Table 1:** The comparisons between the tools of the prediction of oral bioavailability (OB) developed by the other groups and prediction of oral drug bioavailability (PreOB) model.

Number	Model name	Description or examples of the model	Reference
1	Lipinski’s “rule of five”	Qualitatively used to predict the absorption and permeability of drugs	(Lipinski et al., 2012)
2	Quantitative structure–property relationship (QSPR) model	A regression model to predict OB based on a dataset of 591 molecules by applying 85 structural descriptors	(Aller et al., 2009)
3	Rule of thumb (RoT)	Multiple linear regression model for predicting OB with 15 structural descriptors	(Yoshida and Topliss, 2000)
4	Physiologically based-pharmacokinetic (PBPK) approach	A compartmental model of absorption and transit (CAT) to predict the fraction of absorbed dose of different drugs	(Yu, 1999)
5	Prediction of oral drug bioavailability (PreOB)	Integrated the effects of Pgp efflux and P450 metabolism to ensure the accuracy of OB prediction	(Xu et al., 2012)

#### Systematic Identification of Multiple Toxin–Target Interaction (SysTox)

For the novel drug development, many efforts are being devoted to evaluate the toxicity properties of drugs. Due to the vastness of chemical space (toxins) and the diversity of biological systems (targets), the prediction of the toxin–target interface remains difficult. Recently, several novel approaches have been proposed to achieve this goal. For example, a chemical genomics approach that focuses on how similar ligands may interact with similar proteins has been applied to predict novel bioactive compounds of a target (Klabunde, 2010; Yamanishi et al., 2010). In addition, Yu et al. have used the network method to explore ligand–target interactions from high-dimensional biological data (Yu et al., 2012). However, the prediction of toxicity information of a variety of compounds by experimental methods remains difficult, and a systems-level analysis of multiple toxin–target associations is still lacking up to now. Therefore, in our previous study, we established a novel systems toxicology approach SysTox (Zhou et al., 2013) to predict the toxin targets and their related networks, which is based on a large-scale database of 33,800 poison–target interactions through the integration of chemical, genomic, and toxicological information and systems biology technologies. The procedures of SysTox are as follows: 1) a systematic model integrating the extracted chemical and genomic features has been developed to predict the multiple toxin–target interactions with its reliability and robustness estimated by support vector machine (SVM) and random forest (RF) methods. And according to the phenotypic diseases, the qualitative classification of targets has been applied to further explore the biological significance of targets, as well as to validate the robustness of the *in silico* models. 2) As an example, a genome-scale toxin–target–disease network of cardiovascular disease is constructed. 3) The topological analysis of the network is implemented to identify drug targets that are most susceptible to attracting the most critical toxins, as well as to uncover the toxin-specific mechanisms. The advantage of our SysTox approach is that it can be used to predict the toxin–target interactions even for targets with unknown 3D structure. It is worth to note that the toxin–target interaction network can help us to identify new toxins and new target proteins simultaneously and infer novel links from the information of known links. The limitation of the SysTox approach is that the drug targets involving DNA or RNA were not integrated into the model due to the insufficiency of toxin–target information. So the prediction of toxins that target RNA or DNA may be an extension in the following work. The approaches to evaluate the toxicity properties of drugs are listed in **Table 2**.

**Table 2:** The approaches for the prediction of the toxicity properties of drugs.

Number	Model name	Description of the model	Reference
1	A chemical genomics approach	Similar ligands that may interact with similar proteins were used to predict the novel compounds of a target	(Klabunde, 2010; Yamanishi et al., 2010)
2	Network method	Predict ligand–target interactions from high-dimensional biological data	(Yu et al., 2012)
3	SysTox approach	Based on a large-scale database of 33,800 poison–target interactions through the integration of chemical, genomic, toxicological information and systems biology technologies	(Zhou et al., 2013)

#### The Prediction of Half-Life (HL)

The biological half-life of a drug is defined as the time required for the human body to metabolize or eliminate 50% of an initial drug dosage. It is noteworthy that measuring and predicting the half-life of a given drug are important for the safe and accurate dosage of the drug (Berezhkovskiy, 2013). At present, several models were proposed to predict the half-lives of drugs. For example, Sharma et al. have proposed the prediction model for peptide half-life (HLP) in intestine-like environment based on 10mer (HL10) and 16mer (HL16) peptides dataset, which helps in estimating half-lives of peptides relatively rather than in absolute terms (Sharma et al., 2014). With the help of seven machine learning methods and molecular descriptors, Lu et al. have proposed an approach to predict elimination of half-life in humans (Lu et al., 2016). In addition, Turner et al. predicted human half-lives of 20 cephalosporins by integrating constitutional, topological, and quantum-chemical descriptors (Turner et al., 2010). Moreover, Arnot et al. developed two half-life prediction models in humans based on molecular fragments and an automated iterative fragment selection method (Arnot et al., 2014). In summary, most models of prediction of half-life were based on drug structures, while the PreHL model was constructed on only eight molecular descriptors of drugs by principal component analysis (PCA). However, it is difficult and time-consuming to predict the half-life of a specific drug.

In a previous study, we have proposed the PreHL model for TCM injection systems (Yang et al., 2014), which is a systematic decision-making model to predict long or short half-lives of drugs by the C-partial least square (C-PLS) algorithm (Boulesteix, 2004; Kidron et al., 2012). More specifically, the PreHL model was built in three steps: 1) *Dataset collection:* One hundred sixty-nine drugs (injection formulation) with their half-life values, DrugBank ID, chemical name, and Chemical Abstracts Service (CAS) number were collected from DrugBank database (Knox et al., 2011), and they were divided into two subsets: a training set (*n* = 126) used to build the model and an independent test set (*n* = 43) to validate the accuracy of the model. 2) *Descriptor calculation and selection:* Molecular descriptors were first calculated to construct the model, and then 43 objective features were selected based on forward stepwise algorithm. Finally, by PCA, only eight of them were applied for C-PLS modeling process. 3) *Model performance:* For internal validation, the model was evaluated by the leave-one-out (LOO) methodology. Bedsides, external validation was performed by all models. The performance of the model was evaluated by short half-life and long half-life accuracies. For internal validation and external validation, the overall accuracy, long half-life accuracy, and short half-life prediction accuracy are all approximately 85–87%. According to the PreHL model, a half-life higher than 4 h is considered as a satisfactory metabolism property of drugs. Furthermore, the PreHL model was successfully used to assess the half-lives of the potential bioactive components of reduning injection (Yang et al., 2014). The models or approaches of half-life are listed in **Table 3**. Compared with other models, PreHL is a more systematic decision-making model addressing the plasma protein binding, active transport across the membrane, absorption, BBB permeability, drug metabolism, and half-life in the body.

**Table 3:** The models or approaches for the prediction of half-life.

Number	Model name or approaches of half-life	Description of the models	Reference
1	Model of peptide half-life (HLP)	For HLP in intestine-like environment based on 10mer (HL10) and 16mer (HL16) peptides dataset	(Sharma et al., 2014)
2	An approach to predict elimination half-life in human	Seven machine learning methods and molecular descriptors	(Lu et al., 2016)
3	The prediction model of 20 cephalosporins	By the integration of constitutional, topological, and quantum-chemical descriptors	(Turner et al., 2010)
4	Two half-life prediction models in humans	Based on molecular fragments and an automated iterative fragment selection method	(Arnot et al., 2014)
5	PreHL model	The C-partial least square (C-PLS) algorithm	(Yang et al., 2014)

### Identification of Drug Targets

The identification of drug targets of TCM is a basic problem in the processes of drug development, as well as an essential factor for the dissection of the holistic mechanisms of action of TCM (Cao et al., 2012). Currently, the LBVS, SBVS, and the text mining-based approach are widely used to predict the target–ligand interactions. In brief, LBVS aims to identify novel compounds by comparing candidate ligands with the known drugs of a target protein (Byvatov et al., 2003; Krejsa et al., 2003). Nevertheless, if the number of known active compounds for a target is small, the performance of LBVS is poor. In addition, it is difficult to identify drugs with novel structural scaffolds that differ from the known molecules. As for SBVS, it is constrained by the available crystallographic structure of target, thus hampering the prescreening process of drugs. And it is particularly limited for those membrane proteins, like the GPCRs (G-protein coupled receptors), whose 3D structure information is still unavailable up to now (Ballesteros and Palczewski, 2001).

Therefore, to predict the drug–target interactions, we have developed three models, including systematic drug–target identification technology (SysDT) (Yu et al., 2012), weighted ensemble similarity (WES) (Zheng et al., 2015) method, and Pred-binding method (Shar et al., 2016). All the methods of the prediction of drug targets are listed in **Table 4**. In the following part, we will review these methods.

**Table 4:** The methods for the prediction of drug targets.

Number	Model or method name	Description of the model	Reference
1	The ligand-based virtual screening (LBVS)	By comparing candidate ligands with the known drugs of a target protein	(Byvatov et al., 2003; Krejsa et al., 2003)
2	Structured-based virtual screening (SBVS)	Based on the available crystallographic structure of target	(Ballesteros and Palczewski, 2001)
3	Ligand-based approach	Based on the families or subfamilies of targets	(Huang et al., 2013b)
4	Target-based approach	Divide the receptors and pooled together the known ligands into clusters	(Nagamine and Sakakibara, 2007)
5	*In silico* model for predicting the drug–target interactions	By the integration of the amino acid sequences, two-dimensional chemical structures, and mass spectrometry data, as well as the chemical functional groups and biological features	(He et al., 2010)
6	The SysDT model	By the integration of artificial intelligence computing methods systems biology, chemical genomics, and structural genomics, which are based on two powerful methods, random forest (RF) and support vector machine (SVM)	(Yu et al., 2012)
7	Weighted ensemble similarity (WES) method	Based on the theory that the systematic features of ligands that could accurately reflect the ligand–receptor binding pattern	(Zheng et al., 2015)
8	Pred-binding method	Based on 1,589 Dragon descriptors of ligands and 1,080 protein descriptors, by SVM and RF	(Shar et al., 2016)

#### The SysDT Model

The SysDT model was developed as a systematic approach for the prediction of the drug–target interactions that integrated artificial intelligence computing methods systems biology, chemical genomics, and structural genomics, which are based on two powerful methods, RF and SVM (Yu et al., 2012) The model was constructed by 6,707 drugs and 4,228 targets with known drug–target interactions in the DrugBank database, which constructed the positive samples. The negative samples were obtained by three steps: I) re-coupling all drugs and targets in the benchmark dataset into pairs, II) discarding those drug–protein pairs that appeared in the positive samples and keeping the remaining pairs to represent the non-interaction space, and III) randomly selecting the negative pairs from the non-interaction space to ensure the same number as the positive pairs. Then, by SVM, numerical vectors of the drug–target pairs (for both positive and negative samples) by concatenating chemical descriptors and protein descriptors were mapped into a higher dimensional feature space, which is a maximal margin hyper-plane that separates the positive from the negative samples by using a kernel function. Another method, RF, was also used to build a model, which is an ensemble of unpruned classification or regression tree. Finally, the performance of the models was evaluated by internal five-fold cross-validation and four external independent validations with the known drug–target interactions.

Our results showed that the optimal models by SVM showed impressive prediction performance, with a concordance of 82.83%, a sensitivity of 81.33%, and a specificity of 93.62%. Both SVM and RF demonstrate the reliability and robustness of the obtained models. Compared with the structure-based simulation methods, the SysDT approach is not restricted by the 3D structure of targets. More importantly, the advantage of the SysDT model is that it enables to identify the unrelated targets that may share structure similarity of a chemical with ligands. Moreover, it can promote the multi-target drug discovery by recognizing the proteins targeted by a particular ligand. Therefore, the SysDT approach may provide a reliable analysis tool for drug target identification of the herbal molecules on human proteins. Although the SysDT model is effective for the prediction of the drug-target interactions, it is limited by the information of the 3D structure features of the ligand-binding domains. Therefore, novel optimal approaches are still needed to be proposed in further research.

#### The WES Method

**Figure 3:**
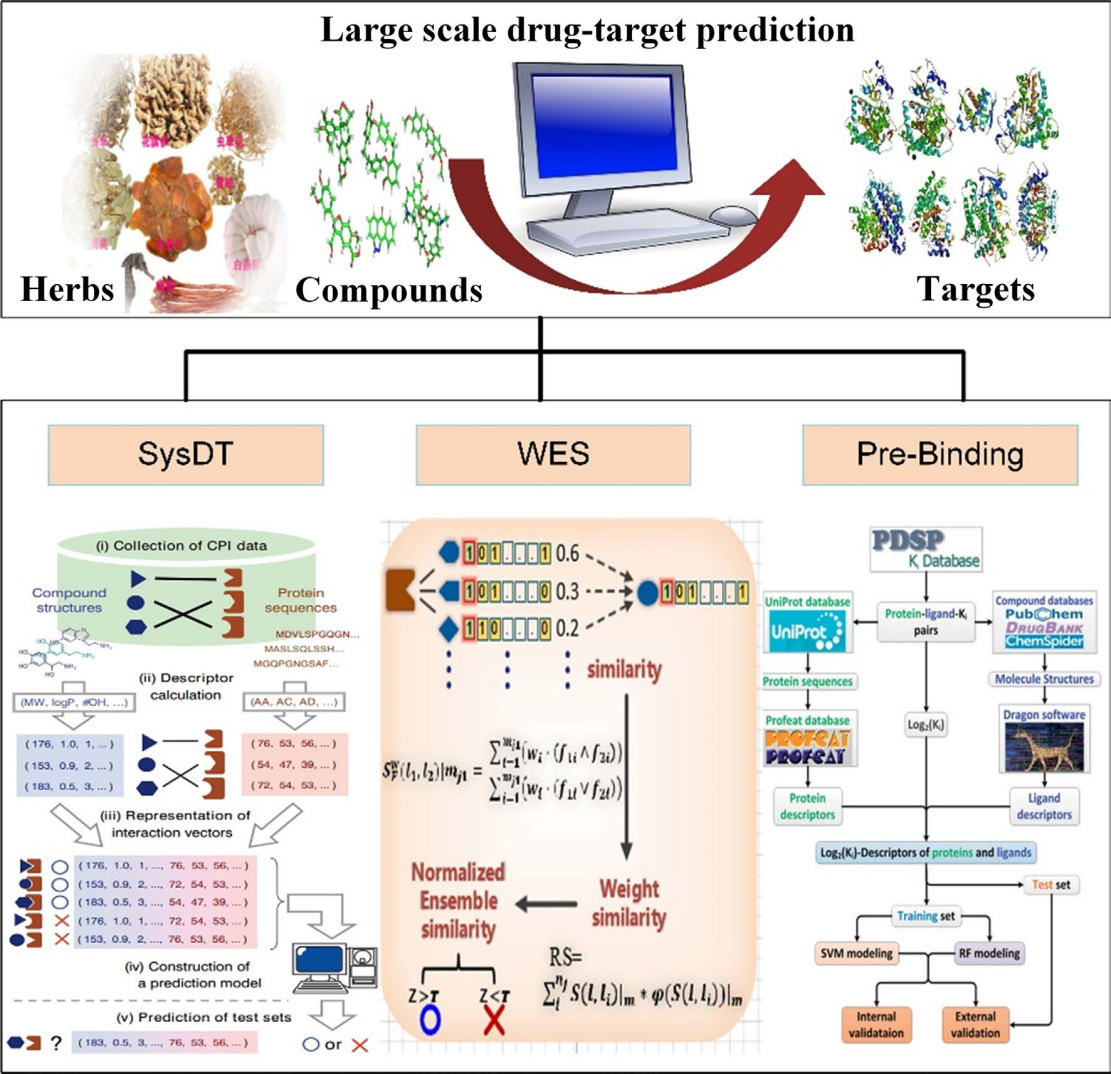
Drug target identification approaches of systematic drug–target identification technology (SysDT), weighted ensemble similarity (WES), and Pred-binding models.

The available computational approaches mainly focus on the prediction of indirect targets of drugs or direct targets of drugs in a small scale. To further improve the drug target prediction systems, we have successfully developed two optical mathematical models: 1) a WES method and 2) a Pred-binding approach (**Figure 3**) to identify the direct targets of drugs based on large scale of drug–target interactions.

The WES approach was proposed on the theory that the systematic features of ligands could accurately reflect the ligand–receptor binding pattern. The WES method was constructed based on over 900,000 drug–target relations, including three steps: 1) identifying the key ligand structural features that strongly related to the pharmacological properties in a framework of ensemble; 2) confirming the targets of drugs by the evaluation of the overall similarity (ensemble) rather than a single ligand judgment; 3) obtaining the overall similarity with the ligand set by integrating the standardized ensemble similarities (*Z* score) by Bayesian network and multi-variate kernel approach; and 4) evaluating and validating the performance of the approach by leave-one-out cross-validation (LOOCV) and the ligand-binding assay test experiments. The WES method exhibits good reliability with a good specificity and sensitivity [Area Under The Curve (AUC) = 0.85] and external [both the binding (positive sample) and non-binding data (negative sample)] and experimental test (ligand-binding assay test) accuracies of 70% and 71%, respectively. Notably, it is able to distinguish the direct binding or indirect binding relationships between drugs and targets, which is of great benefit for drug repositioning and discovery (Zheng et al., 2015).

The advantages of WES includes the following: 1) the structural features based on statistical tests and optimization analysis were integrated into a framework of ensemble to reduce dimensionality of dataset and eliminate data noise. 2) The ensemble concept was proposed to ensure the model to predict the target of the drug based on the drug’s similarity with the whole feature of an ensemble. The one nearest neighbor (1NN) model evaluates the probability of drug targets based only on the maximum similarity to the known ligands of the target. Compared with the 1NN model, WES is better in predicting drug targets for various structurally diverse compounds.

#### Pred-Binding Approach

Drug–target interactions are important for exploring biological activities of these proteins. In fact, some drugs may bind to multiple target proteins and sometimes improperly bind to unwanted off-targets (Wang et al., 2013), leading to severe harmful side effects. Therefore, identifying the satisfactory targets of drugs is an urgent task for drug development. In our previous study, we have developed the Pred-binding model to accurately predict the binding strength between drugs and targets (Shar et al., 2016). The Pred-binding model includes the following: 1) *Dataset construction:* The ligand and target dataset information with known binding affinity abstracted from Psychoactive Drug Screening Program (PDSP) Ki database was used to build the model (Roth et al., 2000). After the exclusion of ligand–target–Ki entries with the repeat number of Ki of more than 70, finally, a dataset consisting of 9,948 ligand–target–Ki pairs was constructed. And 1,589 Dragon descriptors of ligands and 1,080 protein descriptors were obtained for further analysis. 2) *Training set and test set construction:* The dataset was split into training (used to build the model) and test (used to validate the model’s accuracy) sets, and they were randomly split into five subsets with equal number, and one subset was selected as the test set, and the others were considered as the training set. 3) *Model building:* Two *in silico* models based on SVM and RF were proposed to predict the binding affinity. 4) *Model validation:* As mentioned above, first, each subset was selected as the test set, and the other four subsets serve as the training set for validating model. The processes were repeated five times. Second, five external independent validations were performed for all models using different test sets. Third, the comparison of the performance of RF model and SVM model by *F* test was performed. The results showed that the cross-validation coefficient was 0.6079 for SVM and 0.6267 for RF, exhibiting a good potent Ki predictability. In conclusion, the Pred-binding approach may contribute to the prediction of novel potential targets, further guiding the drug development. The limitation of the model is the robust and efficient features; therefore, a better regression model needs to be developed.

In summary, the above three models have provided new approaches for the identification of drug targets, which may benefit the drug design and promote the drug development.

### Drug Combination Prediction and Dynamic Analysis Approach

#### Probability Ensemble Approach (PEA) for the Prediction of Drug Combination

Drug combination has been a promising strategy for the treatment of complex diseases with higher efficacy and fewer side effects than has the single-drug treatment (Zimmermann et al., 2007; Al-Lazikani et al., 2012; Roemer and Boone, 2013). *In vitro* approaches, such as the high-throughput screening method (Borisy et al., 2003; Lehár et al., 2009) and the “multiplex screening for interacting compounds” (MuSIC) (Tan et al., 2012), have been proposed to investigate the synergistic drug pairs. However, these methods are time-consuming and cost intensive. Alternatively, several computational approaches have been developed to identify novel synergistic drug pairs by integrating network analysis and chemical biology data (Chou, 2010; Zhao et al., 2011; Tang et al., 2013). The majority of these methods are limited to dissect the molecular mechanisms or identify combinatorial drugs based on targets with multiple diseases. In addition, some attention has been focused on pharmacokinetic properties of the compound, pharmacodynamic constants, or both pharmacokinetics and pharmacodynamics to predict the drug–drug interactions. But the systematic analysis for predicting the efficacy and side effects of the known or novel drug pairs is still lacking.

**Figure 4:**
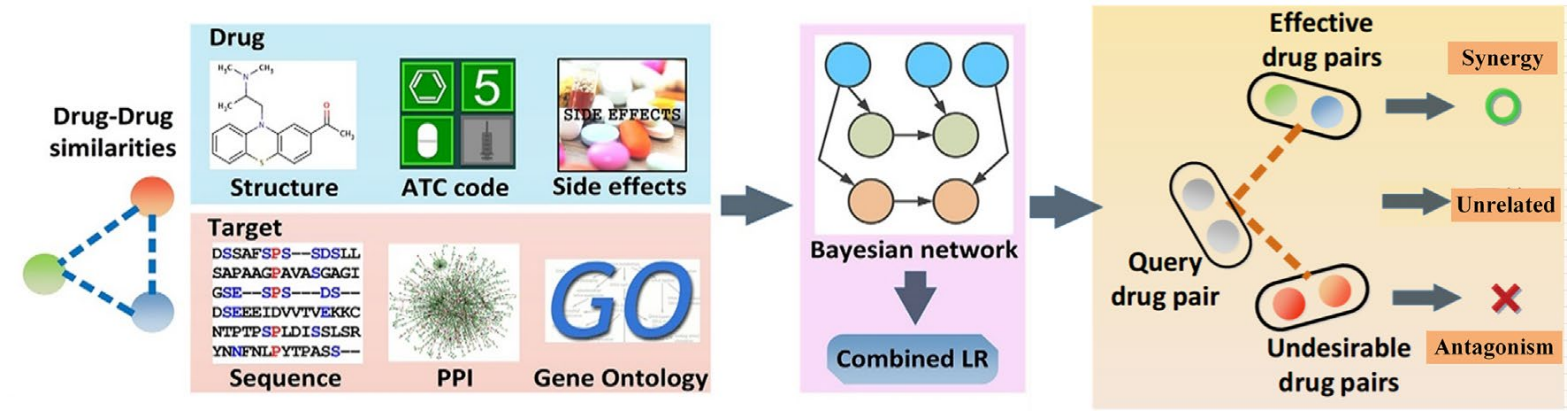
Design processes of the drug combination prediction approach [probability ensemble approach (PEA) model] (Li et al., 2015b).

To clarify the issue, we have proposed the probability ensemble approach (PEA model) (Li et al., 2015b), by the integration of the molecular chemical space, the pharmacological space, the gene annotations, and the biological networks, for the prediction of drug combinations (**Figure 4**). First, by the integration of drug molecular and pharmacological phenotypes, a Bayesian network model based on a similarity algorithm was developed for the prediction of both clinical efficacy and adverse effects. The performance of PEA showed that the combination efficacy of drugs with high specificity and sensitivity (AUC = 0.90), which was further verified by independent data derived from the literature or novel experimental assays. Second, PEA also assesses the adverse effects (AUC = 0.95) quantitatively and predicts the potential therapeutic indications of drug combinations. Finally, the PreDC (Predict Drug Combination) database was constructed with 1,571 known and 3,269 predicted optimal drug combinations associated with their therapeutic indications and potential side effects. In addition, the standalone software and web server of the PreDC are freely available at http://lsp.nwu.edu.cn/predc.php.

Compared with the simple feature-enrich method proposed by Zhao et al. (2011), the PEA algorithm exhibited good advantages with high training efficiency and extensive applicability (the comparison of the methods for the prediction of drug combination is shown in **Table 5**). More particularly, PEA shows similar performances as the whole-feature model by integrating the weakly predictive features, such as target sequence and chemical structure, to improve the performance, making it convenient and easy to understand. Generally, owing to the unknown underlying molecular mechanisms of combination therapies, drug combinations are predicted based on clinical rules derived from clinical experience or randomized clinical trials. Therefore, the drug combinations were predicted only with the similar functions. Notably, PEA has shown that 43% of our high-confidence predictions (with P1 ≥ 0.9 and P2 ≤ 0.1) are predicted as effective drug combinations with different Anatomical Therapeutic and Chemical (ATC) classes (the first level), indicating that PEA is not restrained by the rule. Moreover, PEA model was experimentally validated by 10 novel effective drug combinations that are a combination of antibacterial and anticancer drugs, showing that 80% pairs are synergistic to cancer models. Moreover, the PEA algorithm has incorporated the clinical efficacy and adverse effect evaluation to identify the potential drug combinations effectively. The limitation of the PEA is that the dosage was not integrated into the model; therefore, it should be taken into account to improve the prediction of drug combinations.

**Table 5:** The approaches for the prediction of drug combination.

Number	Approach name	Description	Reference
1	High-throughput screening method	*In vitro* approaches	(Borisy et al., 2003; Lehár et al., 2009)
2	Multiplex screening for interacting compounds (MuSIC)	*In vitro* approaches	(Tan et al., 2012)
3	Several computational approaches	By integrating network analysis and chemical biology data	(Chou, 2010; Zhao et al., 2011; Tang et al., 2013)
4	Simple feature-enrich method	By simple feature-enrich method to predict drug combinations	(Zhao et al., 2011)
5	Probability ensemble approach (PEA model)	By the integration of the molecular chemical space, the pharmacological space, the gene annotations, and the biological networks	(Li et al., 2015b)

#### Network Elementary Subgraphs and Dynamic Modeling Analysis (NetSyner)

TCM is a complex system with multiple compounds and multiple targets; particularly, natural products derived from TCM with weak binding affinity have been proved to have satisfactory therapeutic efficacy through the regulation of the coordination equilibrium of the whole biological network (Zhu and Xu, 2003; Tan, 2007; Huang et al., 2013a). Recently, nearly ∼110,000 small molecules with low binding affinity have been reported in the public database (Liu et al., 2007). However, a suitable strategy to discover the low-binding-affinity molecules is yet to be constructed.

**Figure 5:**
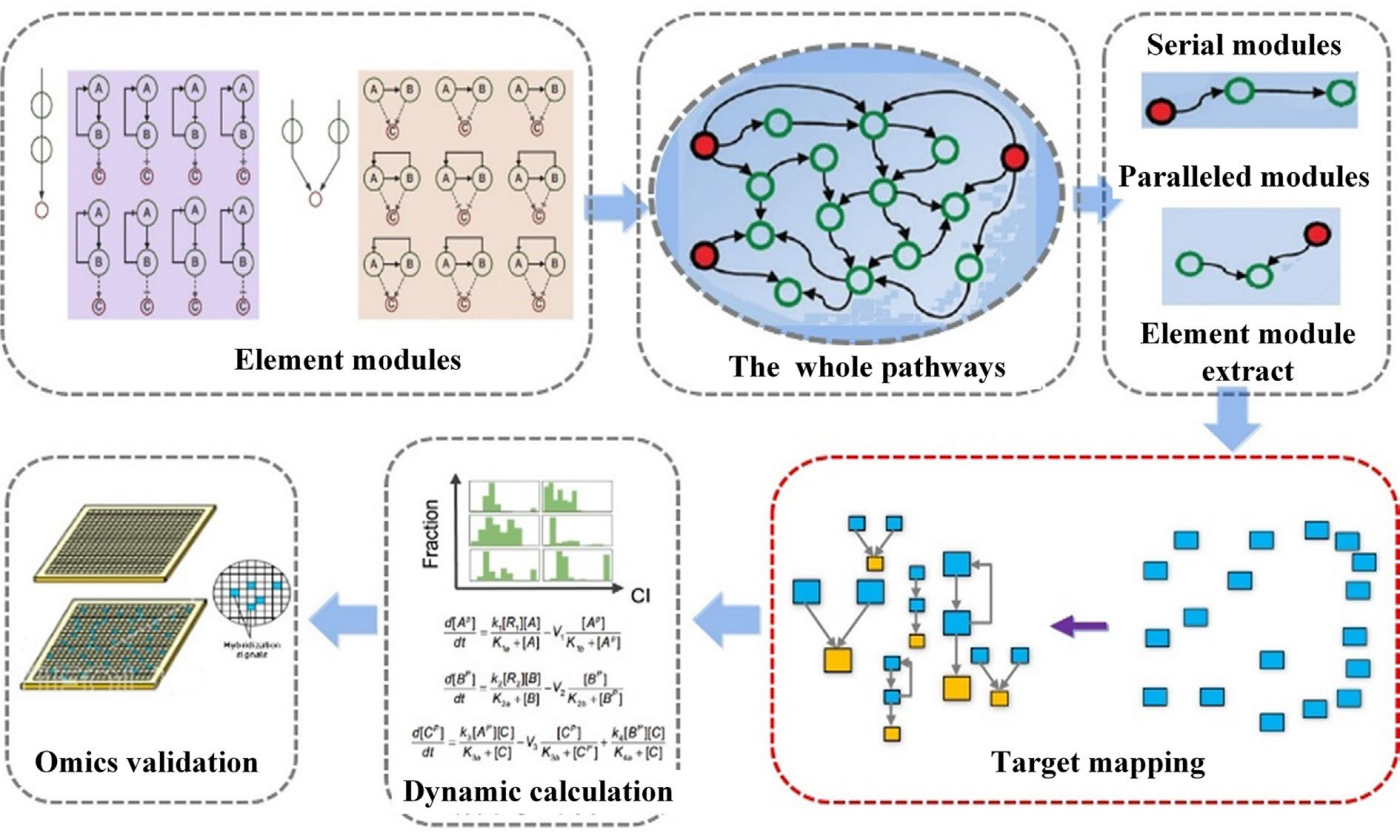
Flowchart of network elementary subgraphs and dynamic modeling analysis (NetSyner) (Wang et al., 2016b).

In a previous study, we have developed a systematic approach NetSyner, which is based on the dynamics of target networks and the dynamics of formula structure to predict the response of perturbation of multiple nodes by cell signaling networks (**Figure 5**) (Wang et al., 2016b). The approach includes three steps: First, dynamic models for a series of three-component elementary subgraphs were built, and 33 elementary subgraphs were performed to determine the desired topology and dynamic parameters among targets. And elementary subgraphs were modeled by a set of ordinary differential equations (ODEs) including the rate laws of mass action and the complete Michaelis–Menten reaction kinetics. The combination index (CI) was used to evaluate whether the two targets in an elementary subgraph can have a synergistic effect. Specially, the mitogen-activated protein kinase (MAPK) pathway is an evolutionarily conserved and well-studied signaling pathway involved in regulating fundamental cellular processes in response to stress and inflammation (Johnson and Lapadat, 2002; Sabio and Davis, 2014). As an example, through the application of the elementary subgraphs to the MAPK pathway, several optimal target combinations were predicted. Then, all the targets of the formula were mapped into the elementary subgraphs; both the modes (synergistic, antagonistic, or unrelated) and extent (synergistic index) of interactions between the bioactive compounds were calculated by the dynamic analysis. Moreover, molecular dynamics simulation and molecular mechanics Poisson–Boltzmann surface area (MM-PBSA) methods were employed to evaluate the binding free energies between the compound and the targets. Furthermore, to experimentally validate the prediction of NetSyner, analyses of the inhibitory effects of the two natural products (luteolin and tanshinone IIA) and the four known selective inhibitors on IL-6 and TNF-α production were carried out. The results indicated that multi-weak perturbations of luteolin and tanshinone IIA against the MAPK signaling pathway can potentially decrease the inflammatory response. In conclusion, weak-binding drugs exhibit favorable efficiency and few adverse reactions, which may offer a promising future for novel drug discovery. Nevertheless, due to the parameter independent model of NetSyner, it is applicable to those pathways that must be satisfied by two conditions: 1) pathways must be evolutionarily conserved and 2) the parameters of the pathway must be intact.

## Application of Systems Pharmacology in TCM

### Construction of TCM Systems Pharmacology Software and Databases

At present, several databases have been established for the investigation of TCM from different aspects. (The database of TCMs were listed in **Table 6**.) For example, TCM Database@Taiwan (Chen, 2011) and TCM-ID (Chen et al., 2010) have provided a large number of herbal ingredients with 3D structures and functional properties. TCMID (Xue et al., 2013) consists of TCM formulae, herbs, ingredients, and their related targets and diseases. Both ChemTCM (Ehrman et al., 2007) and HIT (Ye et al., 2011) focus on herbal ingredients and their corresponding targets. The CVDHD database (Gu et al., 2013) focuses on natural products associated with cardiovascular diseases and targets. But there is lack of systematic network pharmacology analysis among these databases.

**Table 6:** The database of TCMs.

Number	Database name	Description	Reference
1	TCM Database@Taiwan	Provides a large number of herbal ingredients with 3D structures and functional properties	(Chen, 2011)
2	TCM-ID		(Chen et al., 2010)
3	TCMID database	Consists of TCM formulae, herbs, ingredients and their related targets and diseases, drug–target networks, and drug–disease networks	(Xue et al., 2013)
4	ChemTCM	Focuses on herbal ingredients and their corresponding targets	(Ehrman et al., 2007)
5	HIT		(Ye et al., 2011)
6	CVDHD database	Focuses on natural products associated with cardiovascular diseases and targets	(Gu et al., 2013)
7	TCMSP database	Consists of herbs, their chemical molecules, ADME properties, targets, and disease information	(Ru et al., 2014)
8	SymMap database	Focuses on TCM symptoms and their relationships to herbs and diseases	(Wu et al., 2019)
9	ECTM database	Includes the herbs’ basic property and quality control standard, formula composition, ingredient drug-likeness, the gene targets of the ingredients, and related pathways or diseases.	(Xu et al., 2019)

Therefore, our team proposed a unique systems pharmacology platform of TCM-TCMSP (Ru et al., 2014; Liu et al., 2016) (http://lsp.nwu.edu.cn/tcmsp.php). The database consists of more than 36,000 chemical molecules and forms a complete library of Chinese medicine ingredients. In addition, the database integrated 12 ADME key properties like human oral bioavailability, half-life, drug-likeness, Caco-2 permeability, blood–brain barrier and Lipinski’s rule of five, and the drug-likeness analysis of compounds, with more than 4,000 targets and 1,000 types of disease information. More importantly, “drug–target–disease” network pharmacology analysis tools were developed as a novel tool for the identification of the specific targets and the specific diseases of active molecules/groups in TCM. In summary, the particular strengths of TCMSP are the large number of herbal ingredients with ADME properties and their ability to analyze drug–target networks and drug–disease networks, thus providing a platform to dissect the mechanisms of action of TCM, uncover nature of TCM theory, and develop novel herbal-oriented drugs. Moreover, the related software can be used to search the information in the database conveniently. Recently, two novel databases, SymMap (Wu et al., 2019) and ETCM databases (Encyclopedia of Traditional Chinese Medicine) (Xu et al., 2019), were built. SymMap is an integrative database of TCM enhanced by symptom mapping. SymMap is an integrative database, consisting of the information of TCM symptoms and related herbs, diseases and associated symptoms, herbal ingredients, and gene targets. Furthermore, SymMap could be applied to predict component pairwise relationships by statistical tests to filter promising results to guide drug discovery. Actually, SymMap was focused on TCM symptoms and their relationships to herbs and diseases, which provides both candidate leads and screening directions for phenotypic drug discovery. As for the ETCM database, it contains comprehensive and standardized information of 403 TCM herbal species, 3,962 TCM formulae, 7,274 herbal ingredients, 2,266 validated or predicted drug targets, and 3,027 related diseases. ETCM is convenient to obtain the information of the herbs’ basic property and quality control standard, formula composition, ingredient drug-likeness, the gene targets of the ingredients, and related pathways or diseases.

Compared with SymMap, TCMSP is a more comprehensive database that integrated all the herbs and their related compounds, the compound ADME properties, potential targets, and diseases, which can automatically establish the compound–target and target–disease networks to analyze the drugs’ mechanisms of action and promote the TCM drug development. The limitation of TCMSP is that it lacks some medicinal and pharmacological data, the dose–effect relationship of ingredients, and the drug action modes: stimulation or inhibition, drug combination for various diseases, and tissues and organs that the compounds target. To improve these limitations, the ETCM database provides the habitat and quality control information of herbs, which may become a major data warehouse for TCM to promote TCM drug development.

Although tremendous eﬀorts have been made in the past to provide databases containing cancer-related information, to our knowledge, no such dedicated comprehensive repository of anticancer herbs and anticancer herb-originating natural products has been developed currently as yet. Some databases like CancerDR (Kumar et al., 2013) and CancerPPD (Tyagi et al., 2015) have been made in the past to provide comprehensive data involved in anticancer ingredients. However, the CancerDR mainly focuses on FDA-approved and experimental drugs, and CancerPPD is a database of anticancer peptides and proteins. Considering the bleak situation of cancer and absence of systematic database for anticancer herbal products, for the first time, we have developed a comprehensive repository named anticancer herbs database of systems pharmacology (CancerHSP). The CancerHSP database provides information of 2,439 anticancer herbs, 2,439 anticancer active compounds, the molecular structure of each compound, and antitumor activity data based on 492 different cell lines (Tao et al., 2015). Furthermore, the database also consists of natural products with anticancer effects, their related ADME properties, antitumor activity, and target information, which not only helps to dissect the underlying molecular mechanisms of anticancer drugs but also provides basic data support for the development of anticancer drugs.

### Synergistic Effects of the Active Components in TCM

#### Multi-Target Synergistic Effects of TCM

Based on network pharmacological methods, scientists discovered that TCM exhibits multi-target synergistic effects. For example, Violeta et al. have built a computer multiphase pharmacology fingerprint (CPF) based on the Gauss integration screening method (GES) to encode the corresponding multiple target fingerprint atlas of drugs. Besides, the approach successfully found that drugs can interact with multiple targets, which provides a novel method for the discovery of new preclinical and clinical drug candidates (Violeta et al., 2014). In fact, if one drug could act on multiple targets, the drug molecules may exhibit better therapeutic effects through targeting on multiple targets under the synergistic effects (Hopkins, 2007; Hopkins, 2008). Recently, Huang et al. successfully dissected the molecular mechanisms of TCM with multiple targets for the treatment of depression; for example, several antidepressant drugs acted on more than 20 targets (Huang et al., 2013a). In addition, Liu et al. illustrated the mechanisms of action for the herb licorice, and the potential bioactive components were identified by the systems pharmacology. For instance, liquiritigenin, licochalcone B, naringenin, and kaempferol were considered as the bioactive compounds that acted on 22 targets related to cough, including ADRB1 (β-1 adrenergic receptor), ADRB2 (β-2 adrenergic receptor), CALM1 (calmodulin-1), PDE4B (cAMP-specific 3′,5′-cyclic phosphodiesterase 4B), PDE4D (cAMP-specific 3′,5′-cyclic phosphodiesterase 4D), HSP90AA1 (heat shock protein HSP 90-α), HSP90AB1 (heat shock protein HSP 90-β), PPARG (peroxisome proliferator-activated receptor γ), and THRB (thyroid hormone receptor β). The flavonoids, including isoliquiritigenin, liquiritigenin, and liquiritin, exerted synergistic therapeutic effects on thrombosis through the regulation of the proteins F2 (prothrombin), F10 (coagulation factor X), and PTGS2 (prostaglandin G/H synthase 2), which are closely involved in the processes of thrombosis. In addition, licochalcone A and licoisoflavanone acted on the proteins 5-hydroxytryptamine 1A receptor (HTR1A), ADRB1, cell division protein kinase 5 (CDK5), D opioid receptor (OPRD1), GSK3B, and HRH1; therefore, they may exert synergetic effects to achieve anti-ischemic effects to treat ischemic heart disease (Liu et al., 2013a).

Moreover, we identified some novel targets, 5-hydroxytryptamine 2A receptor (5-HT2A) and aldose reductase (AKR1B1), which are associated with diabetes. Also, several bioactive compounds in licorice could target proteins of the nervous system, such as monoamine oxidase type B (MAOB), D2 and D3 dopaminergic receptors, and mitogen-activated protein kinase 10 (MAPK10) (Liu et al., 2013a). Notably, we dissected the detoxification mechanism of licorice; for example, the compounds liquiritin and licochalcone G can target the metalloelastase to destroy bacteria and strengthen the tissue macrophages, thus defending against external invasions. In summary, with the aid of systems pharmacology, we generated a novel perspective for better understanding of single herbal medicine for treating various diseases from the molecular level to the systems level. More importantly, it also explained why licorice is a popular herb, as well as the mechanisms of detoxification of the licorice (Liu et al., 2013a).

#### Multi-Pathway Interactions of Herbs

To comprehensively investigate the interactions between herbal ingredients and their related biological processes, a drug–target–pathway network was generated (Chen et al., 2009). The most important pathways are the cellular signaling pathways, which can interact with each other. In addition, various stimuli appear to activate the same downstream targets, thus exhibiting the same cellular functions. For example, Gong et al. identified alternative pathways based on experimental data, which are involved in regulating cell functions (Gong and Zhang, 2005). In the target–pathway network, targets that appear in multiple pathways are often considered as potential key targets for the treatment of complex diseases.

In addition, by a systematic genetic analysis of 24 types of cancers, scientists found that 67–100% of tumor cells were involved in 12 cellular signaling pathways and related carcinogenesis processes (Jones et al., 2008). Li et al. found that multiple compounds were involved in multiple pathways in Compound Danshen Formula: 58 compounds were associated with the glucocorticoid and inflammatory signaling pathways; 56 compounds acted on the l-arginine/NO signaling pathways; 35 compounds disturbed the renin–angiotensin–aldosterone pathways; and 31 compounds regulated signaling pathways associated with platelet aggregation. Interestingly, all these signaling pathways are closely related to inflammation and coagulation, indicating that Compound Danshen Formula may synergistically regulate these signaling pathways to treat cardiovascular diseases effectively (Li et al., 2012b). Therefore, multi-target drugs of TCM are likely to be involved in alternative pathways or biological processes to treat complex disease effectively instead of single-target drugs.

#### Combinations of Herbal Compounds Acting on Multiple Organs

**Figure 6:**
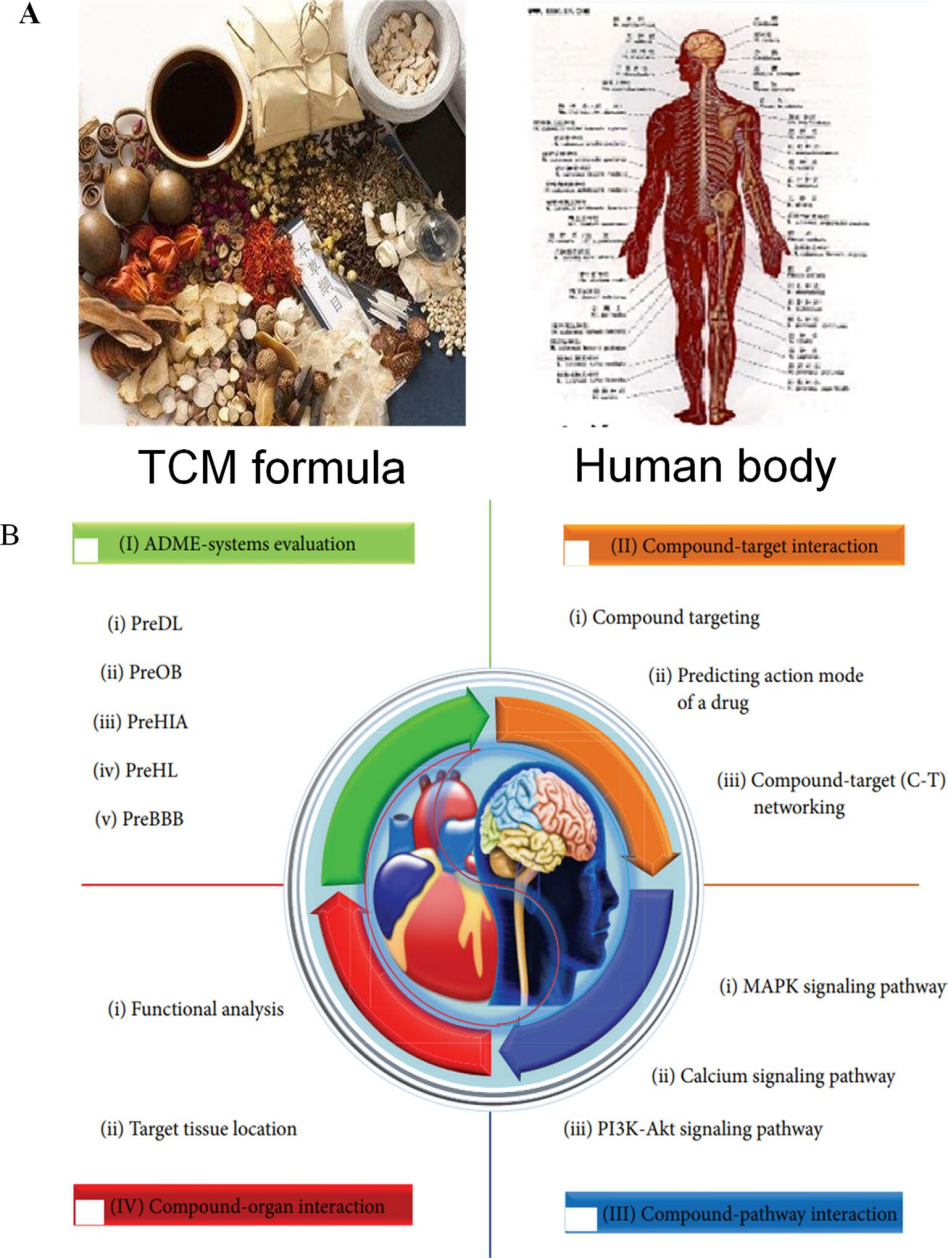
Multi-organ interactions of the multiple compounds in TCMs. **(A)** The holistic herb–human interactions. **(B)** The strategy of systems pharmacology of TCMs for the treatment of complex diseases (Wang et al., 2015).

TCM is a part of holistic medicine, which concentrates on systematic health care for the whole human body rather than one part of the body (**Figure 6A**) (Ventegodt et al., 2014); however, to understand the mechanisms of action of TCM at a systems level is still difficult. Indeed, there are two key problems: 1) If the compound and the person are considered as whole entities, how do they interact with each other? 2) How do the molecules, tissues, and organs of the body respond to different molecules or molecule groups in a formula under holistic frameworks? To solve these problems, in the previous studies, we have examined the molecular basis of some diseases associated with different organs, such as cardio-cerebral diseases and cardiovascular diseases (CVDs) and gastrointestinal disorders (GIDs). The systems pharmacology model consists of four modules (**Figure 6B**): 1) an ADME evaluation model, including oral bioavailability prediction, drug-likeness evaluation, human intestinal absorption, half-life, and BBB permeability prediction; 2) network target fishing and pathway analysis; 3) compound–pathway analysis; and 4) drug–organ enrichment and interaction model (Wang et al., 2015).

Take Xinnaoxin Pill and Sanhe Decoction as examples; with the help of systems pharmacology, we dissected the scientific connotations of simultaneous treatment for cardio-cerebral diseases, and CVDs and GIDs. More specifically, we found that several components in Xinnaoxin Pill exhibited good BBB permeability, suggesting that it may be beneficial for the cardiovascular system. Besides, it could act on several organs involved in multiple biological processes and multiple pathways associated with multiple functions, such as inflammation, myocardial contraction, and angiogenesis, thus allowing the simultaneous treatment of cardio-cerebral diseases.

**Figure 7:**
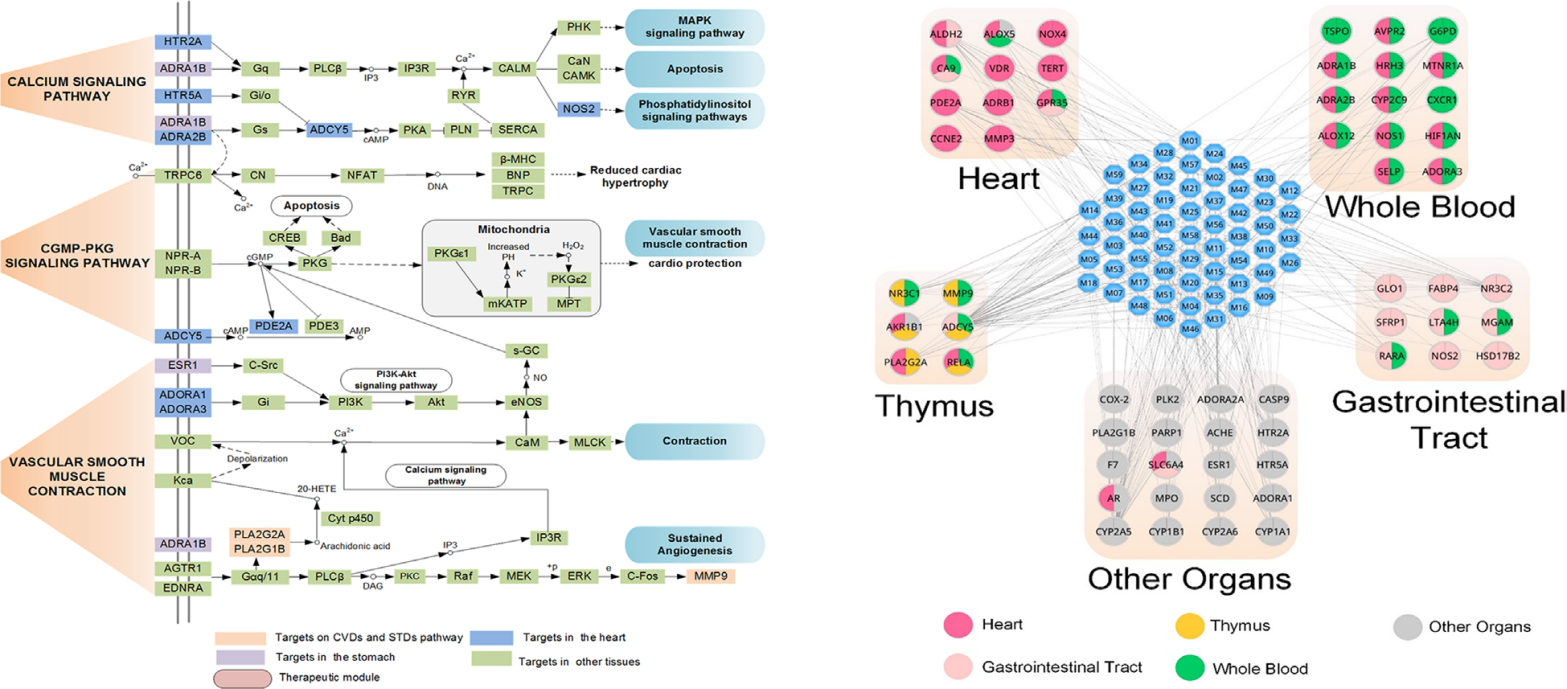
Composition of TCMs has effects on a combination of multiple organs. **(A)** Pathways and therapeutic modules associated with cardiovascular–gastrointestinal diseases. **(B)** The target organ location map; the node represents the organ where the target is located (Zhang et al., 2016).

Moreover, by ADME system evaluation, we identified 59 potential active compounds in Sanhe Decoction (Zhang et al., 2016). Seventy target proteins of these compounds were predicted by target fishing. The compound–pathway network analysis revealed that multiple drugs were simultaneously involved in several pathways, such as calcium ion signaling pathway, cGMP–dependent protein kinase (PKG) signaling pathway, and vascular smooth muscle contractions (**Figure 7A**), suggesting that these drugs tend to exhibit multi-target synergetic or additive effects. The target tissue distribution network indicated that the compounds of Sanhe Decoction acted on multiple tissues or organs simultaneously, the majority of which were associated with heart and stomach, thereby achieving therapeutic effects on CVDs and GIDs (**Figure 7B**). Furthermore, Sanhe Decoction significantly alleviates the myocardial conditions compared with those of the control group in a rat model of myocardial ischemia, verifying the reliability of the theoretical model (Zhang et al., 2016).

In conclusion, the systems pharmacology approach provides a holistic strategy for rational drug design for complex associated diseases, promoting the drug development.

#### Bidirectional Regulation of TCM for the Treatment of Diseases

**Figure 8:**
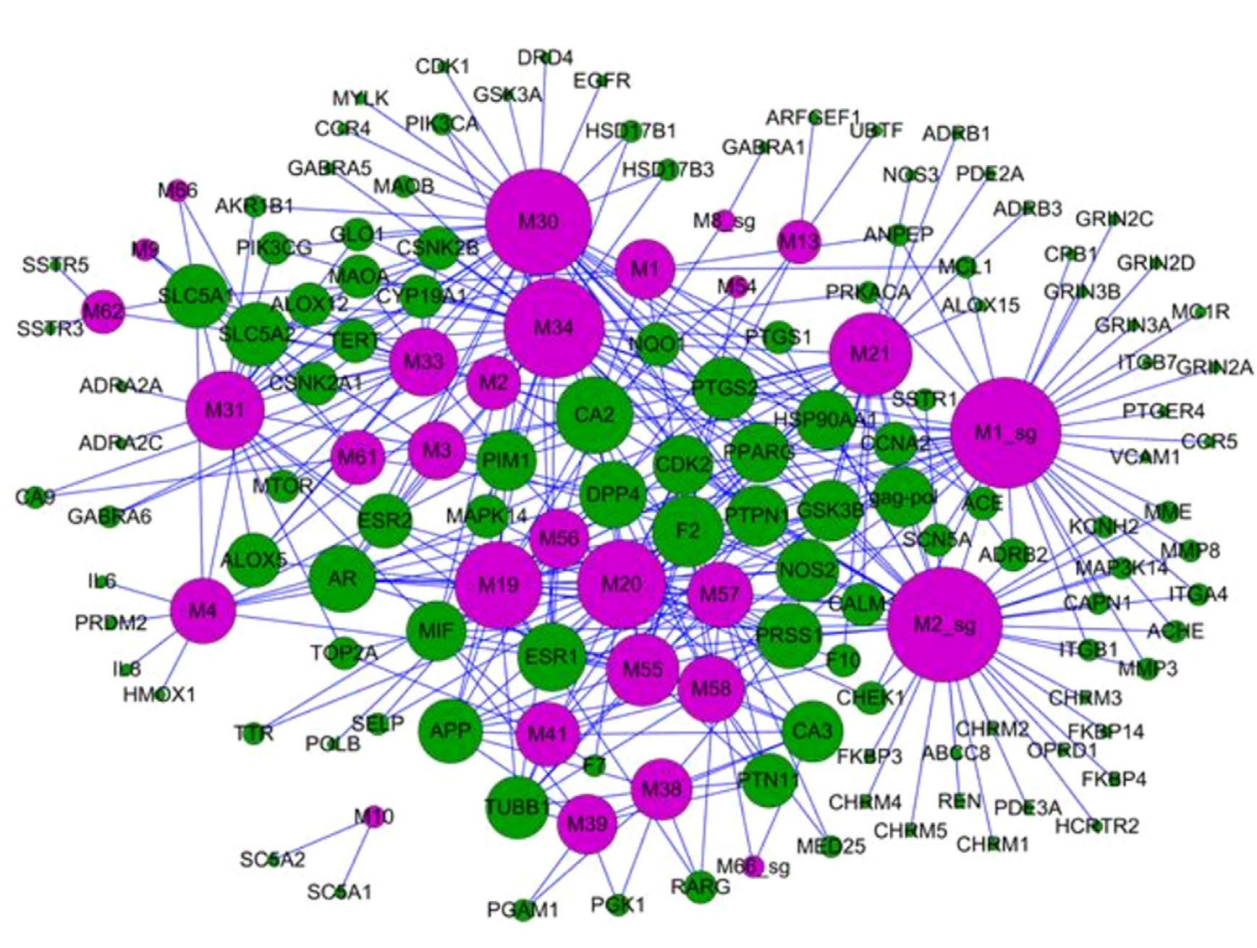
Bidirectional regulation of TCM for the treatment of diseases; the target–disease network of the reduning injection. It is composed of 49 target nodes (round, purple) and 11 disease nodes (square, green), and the size of the circle is the degree of the node (Yang et al., 2014).

Reduning injection, derived from the experience of ancient Chinese medicine doctors, consists of three herbs: *Artemisia annua* L. (genus *Artemisia*, Asteraceae), *Gardenia jasminoides* J.Ellis (genus *Gardenia*, Rubiaceae), and *Lonicera japonica* Thunb. (genus *Lonicera*, Caprifoliaceae), which are mainly used for the treatment of influenza-like diseases, including viral infections, fever, respiratory diseases, and inflammation (Yang et al., 2014). The target network indicated that different diseases may have the same symptoms and can be cured by the same combination of herbs (Lin, 1998). The mechanisms of reduning injection were illustrated by systems pharmacology. The compound–target network of reduning injection is shown in **Figure 8**. We noticed that arachidonate 5-lipoxygenase (ALOX5) is one of the key enzymes in the formation of proinflammatory eicosanoids from arachidonic acid (Albert et al., 2002), which transforms essential fatty acids into leukotrienes (such as leukotriene B4, C4, D4, and E4). Actually, leukotriene B4 is an effective activator of the chemotactic reaction in white blood cells. In the network, ALOX5 is a common pharmaceutical target against various diseases that interacted with several compounds, such as quercetin and luteolin. Moreover, reduning injection might also control the virus infection by directly targeting viral proteins, such as DNA topoisomerase 2-alpha (TOP2A) to inhibit the virus replication (Wang et al., 2012). The cell experiments also showed that the herbal ingredients reduced the inflammatory response through the regulation of inflammatory cytokines and proinflammatory mediators, such as IL-6, IL-8, TNF-α, and COX2. More importantly, the bioactive compounds in reduning can directly kill the virus through the inhibition of virus expression. In summary, the systems pharmacology-based analysis revealed that the dual regulation of reduning injection not only inhibited virus replication but also exerted anti-inflammatory activities to promote body recovery.

### Application of Systems Pharmacology in the Examination of “Jun-Chen-Zuo-Shi” in the Combination Principles of Formula

#### “Jun-Chen-Zuo-Shi” Combination Principle of Mahuang Decoction and Yujin Formula

**Figure 9:**
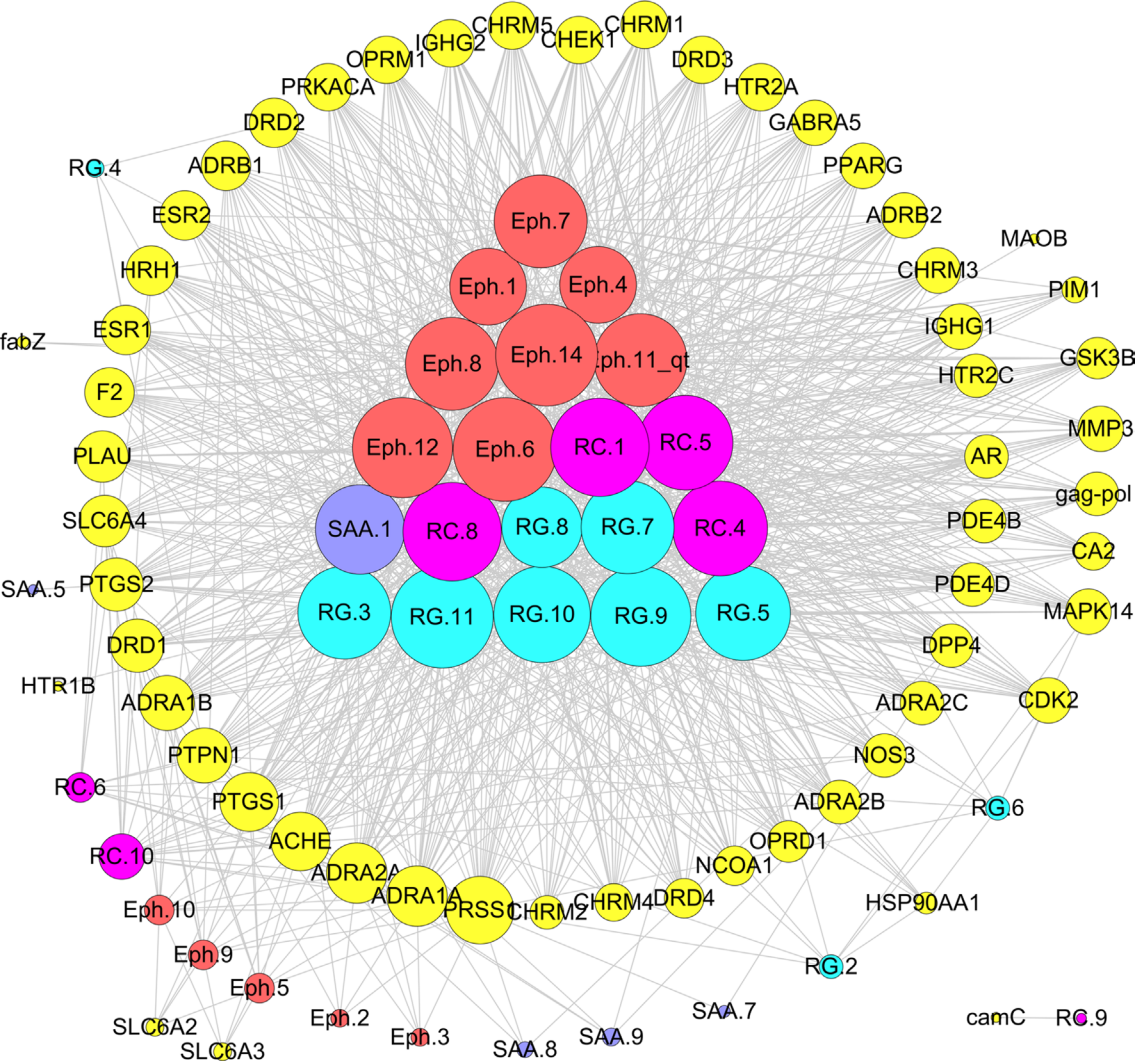
Schematic diagram of the principle of “Jun-Chen-Zuo-Shi” combination principle of Mahuang Decoction. The Eph represents the “Jun” herb ephedra, the RC is the “Chen” (minister) herb cinnamon, the SAA is the “Zuo” (adjuvant) herb almond, and the RG represents the “Shi” (guide) herb licorice (Yao et al., 2013).

“Jun-Chen-Zuo-Shi” is one of the basic principles of herbal formulae. It has been found that there was a clear difference in the structure and biological activity of each ingredient in different herbs and even ingredients in the same herb; however, only some bioactive compounds exhibit therapeutic activities (Zhao et al., 2010). Given the numerous components of a TCM, the interpretation of the rules of combination is difficult. In the previous study, taking Mahuang Decoction as an example, we explored the scientific connotation of the combination principle of TCM (Yao et al., 2013). Mahuang Decoction consists of four herbs: ephedra, cinnamon, almond, and licorice. By the developed systems pharmacology model, the different roles of the four herbs in the prescription were deciphered through the integration of pharmacokinetic interactions, the drug–target network, and the target–disease network from the molecular level to the systems level (**Figure 9**). The main findings were as follows: 1) 45 active compounds were screened by ADME system; among these, 14 potential bioactive compounds belonged to ephedra, including ephedrine, pseudoephedrine, N-methyl ephedrine, and quercetin; 10 compounds were from cinnamon, including cinnamic aldehyde, cinnamic acid, and coumarin; and 9 compounds were from almonds, such as bitter amygdalin and soybean sterol. Licorice has 12 active molecules, which include glycyrrhizic acid, 18-beta-glycyrrhizic acid, and glycyrrhizin; 2) the herb ephedrine plays a prominent role as the “Jun” herb, which mainly stimulates the body heat and asthma through targeting on epinephrine receptor; 3) the “Chen” herb cinnamon can act on the same targets as the “Jun” herb ephedrine, which enhances therapeutic effects. For example, the herb cinnamon also acted on both the beta 1-adrenergic receptor and the beta 2-adrenergic receptor, thus reducing the dose of the “Jun” herb ephedrine required. 4) The “Zuo” and “Shi” herbs almond and licorice helped to improve the bioavailability of the “Jun” and “Chen” herbs and to coordinate all the drug activities to promoting synergistic effects of four herbs.

**Figure 10:**
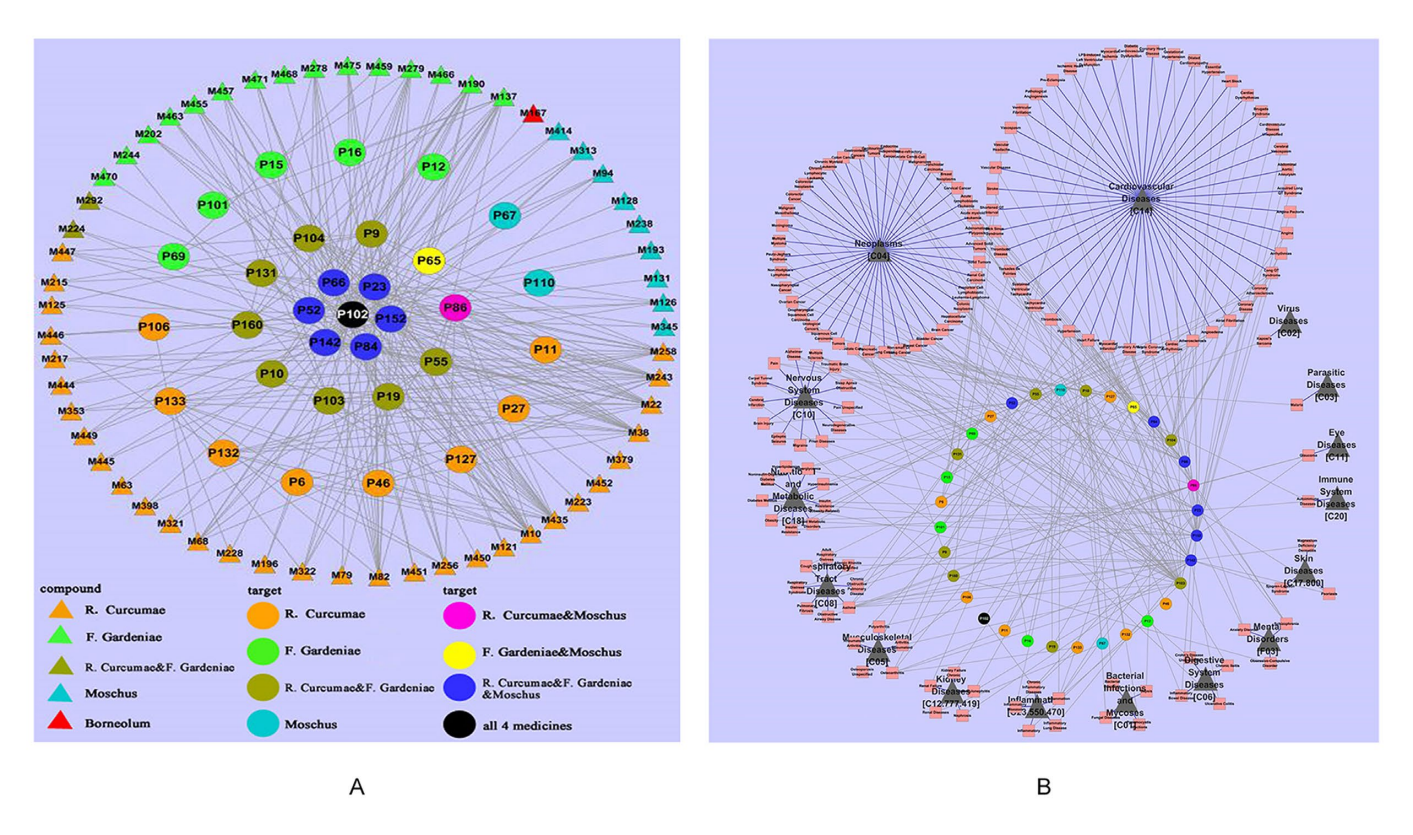
Dissection of the “Jun-Chen-Zuo-Shi” combination principle of Yujin Formula. **(A)** The potential molecule–target networks constructed by 58 potential active components (triangles) and 32 potential targets associated with cardiovascular diseases (CVDs) (round). **(B)** Target–disease network, linked by 32 potential targets (the middle circles were marked with a variety of colors, as in **Figure 2**) and 147 kinds of diseases (red squares), which were divided into 16 types (black triangles) (Li et al., 2012a).

Moreover, we have dissected the famous prescription Yujin Formula for treating cardiovascular diseases to clarify the “Jun-Chen-Zuo-Shi” combination principle in Chinese medicine (Li et al., 2012a). From the Yujin Formula, 58 potential bioactive compounds were identified by ADME screening. The compound–target network indicated that the “Jun” herb *Curcuma aromatic* possessed the most bioactive compounds, which acted on the targets associated with CVDs; the “Chen” herb *Fructus Gardeniae* has fewer bioactive compound and targets and shared 15 targets with the “Jun” herb *C. aromatic* to enhance the therapeutic effects; both the “Zuo” “Shi” herbs musk and borneol play assistant roles by decreasing the toxicity and targeting the ingredients to corresponding organs. In the Yujin Formula, target–disease network (**Figure 10B**) showed that most targets were associated with CVDs (44/147); moreover, they were distributed in tumors (40/147), neurological diseases (13/147), and nutritional metabolic diseases (9/147). These results indicated that Yujin Formula may be applied not only for the treatment of CVDs but also for tumors, nervous system diseases, nutritional or metabolic disease, and other diseases. In summary, the scientific connotations of the “Jun-Chen-Zuo-Shi” combination principle were illustrated, which are of great significance for understanding the mechanisms of TCM.

#### Pathogenesis of Vitiligo and Its Treatment by Qubaibabuqi Formula

Vitiligo is an acquired, pigmentary skin disease that is disfiguring and difficult to treat. Clinically, many TCM prescriptions possess significant effects on vitiligo. Previously, we examined the potential pathogenic mechanisms of vitiligo and its treatment by Qubaibabuqi formula by the systems pharmacology (Pei et al., 2016). Fifty-six active ingredients were identified as the active compounds, including buritin, bubonin, kaempferol, and cholesterol, which played important roles in the treatment of vitiligo. They acted on 83 target ADCY1 (adenylate cyclase type 1), SCD (stearoyl-coenzyme A desaturase), and BCHE (butyrylcholinesterase) to enhance immune response, increase melanin synthesis, and equilibrate the nervous system. In addition, the analysis of the target network and integration of vitiligo pathways showed that the Qubaibabuqi formula may be involved in modules such as immune-related modules, nervous system-related modules, and melanin synthesis-related modules, exhibiting synergistic effects on vitiligo. The study systematically analyzed the potential molecular mechanisms of Qubaibabuqi formula and pathogenesis of vitiligo from the molecular, network, and pathway levels, deepening our understanding of vitiligo and extending the application of TCM in modern medicine.

## Dissection of Syndrome Differentiation Theory and Qi-Blood Theory

TCM is derived from ancient medical practices that integrate the integrity of the body and the natural environment. The concept of entirety and the method of treatment with syndrome differentiation in TCM is distinctive, which provides a basis for the diagnosis and treatment of diseases (Jiang et al., 2012). More importantly, syndrome differentiation has always been an important pharmacological principle to guide the prescription. For example, Liuwei Dihuang Pill and Jinkui Shenqi Pill were developed under the guidance of the syndrome. However, owing to little evidence of the link between diseases and efficacy, the therapeutic strategies under syndrome are still lacking.

With the aid of systems pharmacology, the “drug–gene–targets–disease subtype” network associated with CVDs was established. Therein, the drugs, targets, and multi-level interactions were illuminated, and the complex interactions between disease genes and CVDs’ subtypes were discovered (Li et al., 2014). To uncover the biological basis of CVDs’ syndrome, “CVDs syndrome of qi stagnation, blood stasis, qi deficiency, and blood deficiency” were implemented. Combined with the related TCM and refined prescription, the “syndrome–gene–target–drug” network was established to clarify the molecular network and pathways in coronary heart disease with the characteristic of qi stagnation and blood stasis (Zhou and Wang, 2014).

**Figure 11:**
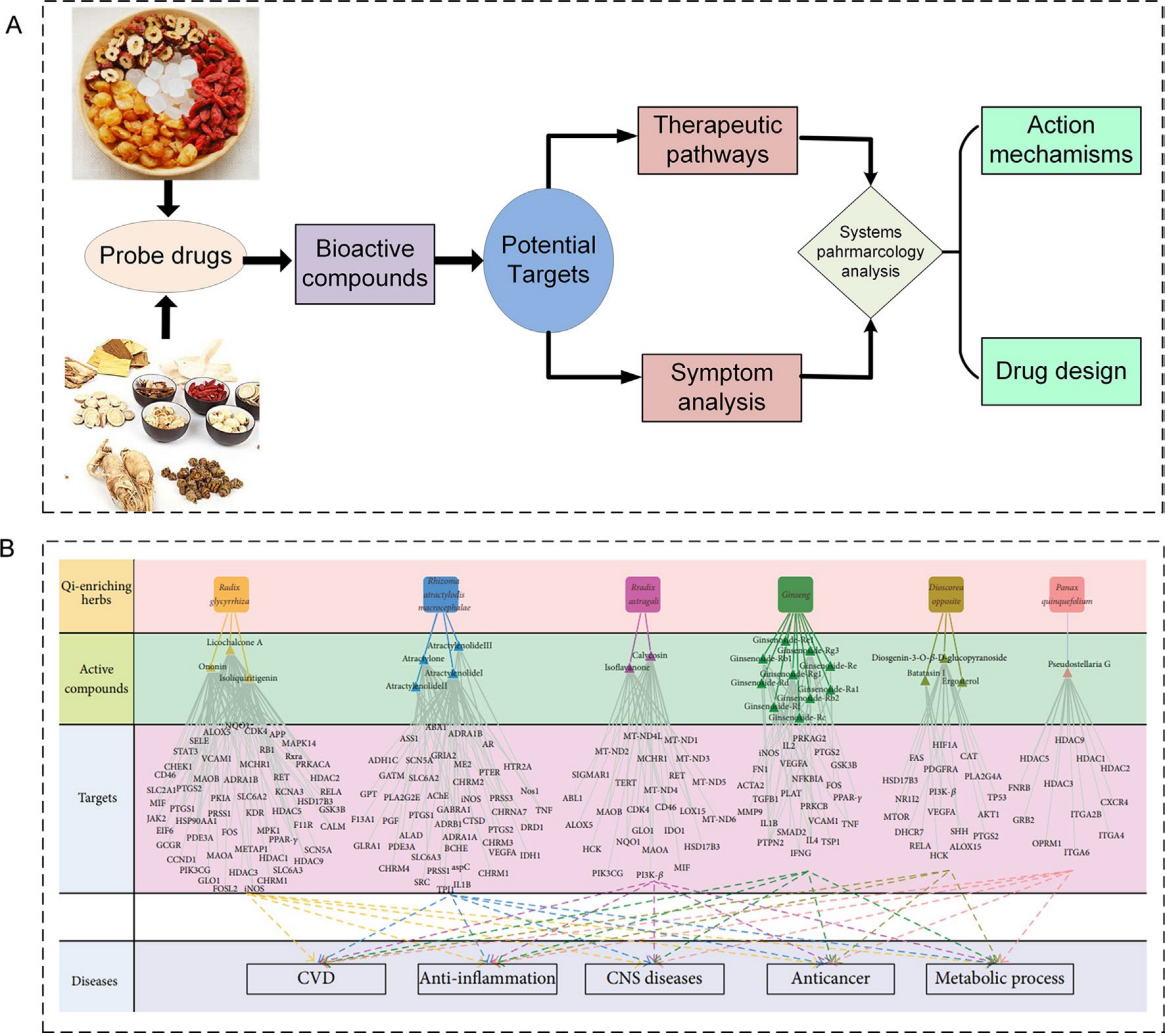
Study of the mechanism of disease and syndrome and the theory of qi and blood. **(A)** The relationship at the molecular level between the different subtypes of cardiovascular disease and the distribution of targets on the pathway; blue indicates blood stasis syndrome, and red indicates qi deficiency syndrome. **(B)** Study of the material basis of qi and bloods (Liu et al., 2013b).

Furthermore, we identified that the qi-tonifying medicines were involved in the enhancement of immunity, the promotion of energy metabolism, and blood circulation, whereas blood-tonic Chinese herbs tended to improve and promote the function of hematopoietic stem cells (**Figure 11**). A computational method was built to distinguish the molecular characteristics of qi-tonifying and blood-tonic molecules, with a prediction accuracy higher than 80%, providing a new tool for the material-based analysis of qi-blood theory and the discovery of new drugs (Liu et al., 2013b).

## Conclusions and Prospects

TCM is a complex mixed system with multiple components and multiple targets; thus, the identification of the potential bioactive molecules and the dissection of the underlying mechanisms of action to establish the optical drug combinations are the essential tasks of TCM. Fortunately, the advent of systems pharmacology framework provides powerful tools for TCM studies: 1) new methods for identification of active components/groups of TCM from the whole perspective. More than 10 mathematical models, including PreOB and PreHF, have been developed, which overcome the limitation of TCM in pharmacokinetic and pharmacodynamic experiments, providing convenient approaches for the discovery of effective substances; 2) large-scale target prediction systems of TCM, with three approaches (SysDT, WES, and Pred-binding) as new tools for drug target discovery; 3) the probability ensemble approach (PEA) model as a novel tool for the dissection of mechanisms of action and the prediction of new indications of TCM; and 4) a novel network of elementary subgraphs and a dynamic model was proposed for the large-scale screening of weak-binding compound in TCM.

With the aid of the systems pharmacology method above, we have built a systems pharmacology database and analysis platform for TCM, which has been applied for the illustration of the synergetic effects of drug combinations, the synergetic effects of multiple targets, pathways and organs, and the bidirectional regulation of Chinese medicine. Moreover, the systems pharmacology of TCM provides methodological guidance for the dissection of the combination principle and syndrome differentiation of herbal formulae, as well as the interpretation of the qi and blood basis of TCM from the molecular level to the systems level. It is of great significance to both the modernization of TCM and the development of modern medicine.

Although the systems pharmacology approach has achieved certain applications and results, the theory and methods require further improvements in the future; for example, the dose of herbs should be added into the model because the efficacy of the same herb obviously differs with different dosages. Therefore, it is necessary to integrate the drug dosage into the systems pharmacology models to provide guidance for clinical applications and further validation. In addition, the systems pharmacology models were constructed predominantly based on computer predictions; however, the reliability and validity of these models still need to be verified by experiments and clinical practice. Besides, the quality of TCM is one of the most important factors for modernization of Chinese medicines, so the study on its genuineness is of great significance for the efficacy of TCM. How to assess the quality of TCM and integrate it into the in silico model should be considered. Furthermore, the development of precision medicine, TCM combination, or a combination of TCM and Western medicine (WM) has obvious advantages. Mass clinical data showed that the complementary advantages of combined TCM and WM can significantly improve the efficacy of treatment for many diseases and contribute to the development of precision medicine (Wang and Zhang, 2017). Therefore, how to predict the drug combination of these TCM and WM and assess the efficacy and side effects is valuable for novel drug design. At present, RCTs have been generally used to assess the clinical efficacy of TCM (Hu et al., 2017). For example, a meta-analysis of RCTs has shown that TCM significantly improved analog scale, Western Ontario and McMaster Universities Osteoarthritis Index (WOMAC), and total effectiveness rates of knee osteoarthritis. In addition, TCM showed a lower risk of adverse events than did standard western treatments (Chen et al., 2016). Studies have shown that TCM is effective in treating atrial fibrillation and has relatively few side effects, but the mechanism of action is still unclear (Wang et al., 2011; Liu et al., 2014; Cai et al., 2017). However, respective RCTs of TCM are limited, because there has been no English meta-analysis of TCM treatment for some diseases.

In the future, we will further improve systems pharmacology and provide extended guidance for the modernization of Chinese medicine and the development of new drugs.

## Author Contributions

WZ wrote the first draft of the manuscript. YH drew the figures. ZM, AQ, and YW helped revise the manuscript.

## Funding

This work was supported by Fund of the Fundamental Research Funds for the Central Universities (no. 3102017OQD050), China’s Post-doctoral Science Fund (no. 2017M623249), the National Natural Science Foundation of China (no. 31570940), and the Key Research and Development Project of Shaanxi Province (no. 2018SF-363).

## Conflict of Interest Statement

The authors declare that the research was conducted in the absence of any commercial or financial relationships that could be construed as a potential conflict of interest.

## Abbreviations

TCM, traditional Chinese medicine; ADME, absorption, distribution, metabolism, and excretion; SOD, superoxide dismutase; CK, creatine kinase; cAMP, cyclic adenosine monophosphate; cTnI, cardiac troponin I; ALOX5, arachidonate 5-lipoxygenase; TOP2A, topoisomerase 2-alpha; ADCY1, adenylate cyclase type 1; SCD, stearoyl-coenzyme A desaturase; BCHE, butyrylcholinesterase.
